# Influence of Wobbling Tryptophan and Mutations on PET Degradation Explored by QM/MM Free Energy Calculations

**DOI:** 10.1021/acs.jcim.4c00776

**Published:** 2024-09-30

**Authors:** Anna Jäckering, Marc van der Kamp, Birgit Strodel, Kirill Zinovjev

**Affiliations:** † Institute of Theoretical and Computational Chemistry, Heinrich Heine University, Universitätsstr. 1, 40225 Düsseldorf, Germany; ‡ Institute of Biological Information Processing: Structural Biochemistry (IBI-7), Forschungszentrum Jülich, Wilhelm-Johnen-Straße, 52428 Jülich, Germany; ¶ School of Biochemistry, University of Bristol, University Walk, Bristol BS8 1TD, United Kingdom; § Departament de Química Física, Universitat de València, 46100 Burjassot, Spain

## Abstract

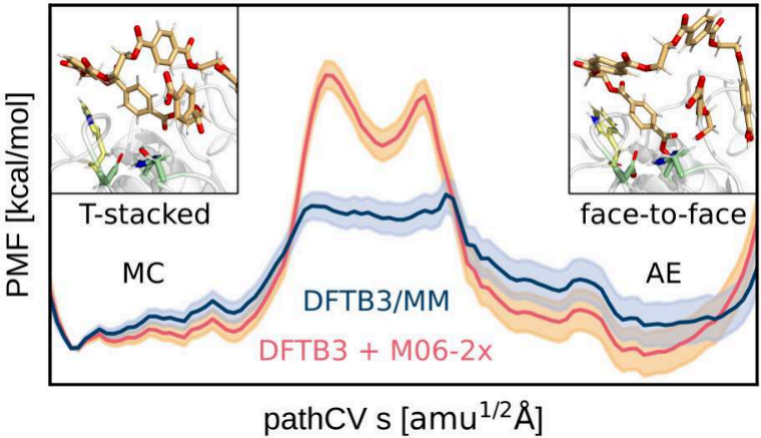

Plastic-degrading enzymes, particularly poly(ethylene terephthalate) (PET) hydrolases, have garnered significant attention in recent years as potential eco-friendly solutions for recycling plastic waste. However, understanding of their PET-degrading activity and influencing factors remains incomplete, impeding the development of uniform approaches for enhancing PET hydrolases for industrial applications. A key aspect of PET hydrolase engineering is optimizing the PET-hydrolysis reaction by lowering the associated free energy barrier. However, inconsistent findings have complicated these efforts. Therefore, our goal is to elucidate various aspects of enzymatic PET degradation by means of quantum mechanics/molecular mechanics (QM/MM) reaction simulations and analysis, focusing on the initial reaction step, acylation, in two thermophilic PET hydrolases, LCC and PES-H1, along with their highly active variants, LCC^IG^ and PES-H1^FY^. Our findings highlight the impact of semiempirical QM methods on proton transfer energies, affecting the distinction between a two-step reaction involving a metastable tetrahedral intermediate and a one-step reaction. Moreover, we uncovered a concerted conformational change involving the orientation of the PET benzene ring, altering its interaction with the side-chain of the “wobbling” tryptophan from T-stacking to parallel π–π interactions, a phenomenon overlooked in prior research. Our study thus enhances the understanding of the acylation mechanism of PET hydrolases, in particular by characterizing it for the first time for the promising PES-H1^FY^ using QM/MM simulations. It also provides insights into selecting a suitable QM method and a reaction coordinate, valuable for future studies on PET degradation processes.

## Introduction

Synthetic polymers, commonly referred to as plastics, have gained significant attraction in the global market due to their durability, affordability, and versatility.^1,2^ Presently, degradation methods for the immense amount of accumulated plastic waste are both environmentally and economically costly, pressing the need for an eco-friendly and efficient alternative.^3^ Enzymatic degradation emerges as a promising solution, particularly focusing on poly(ethylene terephthalate) (PET), a widely used plastic in packaging and textiles.^4,5^ The discovery of enzymes capable of PET degradation in 2005^6^ and the identification of *Ideonella sakaiensis* as an organism metabolizing PET^7^ in 2016 have paved the way for enzymatic degradation.

Enzymes with PET-degradation function are often found among hydrolases (EC 3.1.1.x) and specifically cutinases (EC 3.1.1.74), but are now often categorized into the novel class of polyester hydrolases (EC 3.1.1.101).^7−9^ These enzymes share an α/β-hydrolase fold characterized by a core comprising eight β-strands and six α-helices with a surface-exposed catalytic triad typically composed of serine, aspartate, and histidine, facilitating PET hydrolysis.^10,11^ Despite some wild-type (WT) enzymes exhibiting high activities, enzyme engineering is necessary to enhance efficiency and stability to facilitate industrial application. This has yielded two of the currently most active PET-degrading enzymes, the leave-branch compost cutinase F243I/D238C/S283C/Y127G (LCC^ICCG^) and polyester hydrolase I L92F/Q94Y (PES-H1^FY^) variants.^5,12,13^ Notably, these two variants share a mutation at the same position, yet with divergent effects: activity of LCC could be enhanced by elimination of tyrosine (Y127G) in addition to other mutations present in the LCC^ICCG^ variant, while an introduction of tyrosine (Q94Y) in combination with introduction of phenylalanine in close proximity yielded the PES-H1^FY^ variant with increased activity.^5,13^ We previously found that the entry of PET into the active binding cleft and the subsequent Michaelis complex (MC) formation is promoted by the LCC F243I/Y127G (IG) variant^14^ but the precise effect remains unclear and demands elucidation of the energy profile of the first reaction step, acylation, to fully understand MC stabilization.

**Figure 1:**
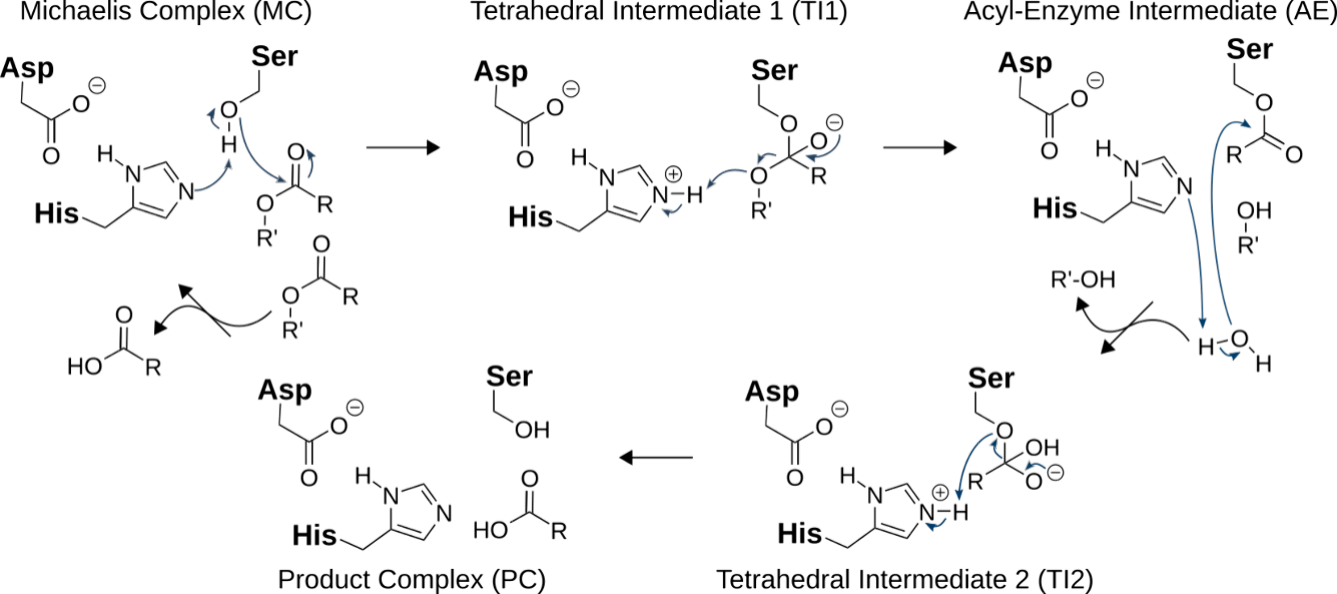
Mechanism of PET degradation as proposed by Boneta et al.^15^ The mechanism can be divided into two steps: First, the acylation of the Michaelis complex (MC) yielding the acyl-enzyme intermediate (AE), and second, the hydrolysis yielding the product complex (PC), both incorporating a tetrahedral intermediate (TI). This study focuses on the acylation step leading from MC to AE, as shown in the top row.

The catalytic hydrolysis reaction of several PET-degrading enzymes was previously investigated using quantum mechanics/molecular mechanics (QM/MM) simulation studies, which enable investigation of the region contributing to the target reaction at the QM level for bond breaking and formation, while managing the computational demand by treating the remaining part of the system at the MM level.^15−17^ These studies revealed two predominant binding poses of PET: pro-*S*^18−20^ and pro-*R*.^5,21^ An energetic preference for catalysis starting from the pro-*S* pose is proposed, which allows for T-stacking π–π interactions between PET’s benzene ring and a conserved tryptophan positioned at one end of the catalytic binding cleft, which is also called “wobbling” tryptophan (LCC: W190, PES-H1: W155).^15,22^ The catalysis mechanism after substrate binding can be divided into two main processes: acylation and deacylation (Figure 1).^15−17,23−26^ During acylation, the catalytic serine’s nucleophilicity increases via proton transfer to the catalytic histidine, which is polarized by the catalytic aspartate. This facilitates nucleophilic attack on the ester’s carbonyl carbon, with the resulting oxyanion stabilized by the oxyanion hole.

Subsequent cleavage of the ester bond yields the acyl-enzyme intermediate (AE), releasing an alcohol molecule from the active site after a proton transfer from the catalytic histidine to the resulting oxyanion. In deacylation, a water molecule’s oxygen nucleophilically attacks the carbonyl carbon of AE, generating a second tetrahedral intermediate (TI) and reprotonating the catalytic histidine. Finally, the AE bond breaks, yielding the second product with a carboxyl group, while the serine is reprotonated, restoring the initial state. Products comprise smaller PET oligomers, as well as mono(2-hydroxyethyl) terephthalic acid (MHET), bis(2-hydroxyethyl) terephthalic acid (BHET) and the final monomers terephthalic acid (TPA) and ethylene glycol (EG).^27−29^

There is debate over whether acylation and deacylation occur in one or two steps, with the TI potentially representing a tetrahedral transition state (TS).^15,16^ Identifying the rate-determining step remains challenging, with some studies suggesting acylation and others deacylation.^15,17,23−26,30−32^ Product diffusion from the active site, necessary for subsequent substrate hydrolysis, may also be rate-limiting, as suggested by Shrimpton-Phoenix et al.,^31^ though others contest this notion.^26^ Various studies discuss the catalytic histidine’s polarization mechanisms, considering proton transfer in addition to pure polarization.^20,25,31^ QM/MM studies highlight the role of specific amino acids (Ser, Ile, Ala) near the catalytic aspartate in stabilizing the histidine, influencing proton transfer dynamics.^23,33^ Mutation experiments confirm the importance of isoleucine, with alanine substitutions resulting in activity loss.^23^ Such insights are crucial for evaluating mutation sites, particularly in enzyme redesign aiming to lower the free activation energy by stabilizing transition states, beyond substrate binding.^34,35^

Another interesting residue is a conserved tryptophan, the “wobbling” tryptophan, which exhibits three different conformations in the mesophilic *Ideonella sakaiensis* PETase (*Is*PETase), while maintaining a single conformation in other, mostly thermophilic polyester hydrolases.^20,36^ This rigidity is attributed to steric hindrance by a nearby histidine, which in *Is*PETase is substituted by a serine. Extensive MD and metadynamics simulations, coupled with experimental analyses, have revealed that the presence of this serine and an adjacent isoleucine in *Is*PETase, instead of histidine and phenylalanine in other PET-degrading enzymes, enhances the flexibility of the tryptophan and loop regions within the active site, positively impacting activity.^37,38^ Moreover, the conformational flexibility of tryptophan allows T-shaped π–π interactions with PET’s benzene ring, thereby expanding the space within the active site and favoring PET binding. Following hydrolysis, suggested conformational changes of the PET benzene ring lead to energetically less favored face-to-face stacking and promote product release.^20,36,38^

While it was found that the conformational flexibility of the “wobbling” tryptophan in *Is*PETase is important for the PET degradation activity,^32^ the detailed impact of the “wobbling” tryptophan on the reaction energetics and the PET conformation remains unexplored. Moreover, prior publications propose inconsistent conclusions regarding whether the acylation mechanism occurs as a one- or two-step process.^15−17,26^ Here, we investigate these two issues in relation to the acylation reaction, and not directly on whether acylation or deacylation is rate-determining (which may be enzyme dependent^17^). We aim to elucidate the conformational change of the benzene ring of PET and its interaction with the “wobbling” tryptophan on the acylation reaction, and distinguish between the possible one- or two-step mechanisms. By investigating two variants of two PETases, we further can highlight whether the more efficient variants show a reduction in the free energy barrier for acylation.

To this end, we performed QM/MM simulations of the acylation step of PES-H1 and LCC as well as their variants, PES-H1^FY^ and LCC^IG^, the latter representing LCC^ICCG^ excluding the stabilizing disulfide bridge. This is the first QM/MM investigation of PES-H1, and we provide additional insights into the LCC, which has been analyzed in several previous studies and thus allows for comparison and validation of our results.^15,17,22,24,33^ The QM/MM method is used in combination with the adaptive string method (ASM),^39^ which offers a flexible approach for incorporating collective variables to delineate the reaction pathway. This methodology sheds light on the previously overlooked impact of the “wobbling” tryptophan, addressing gaps in our understanding of PET degradation mechanisms, thus helping to drive the development of industrially applicable enzymes.

## Methods

### Parameterization

The parametrization of a small PET analogue, comprised of three units, has been previously documented by Pfaff and colleagues.^13^ To model the analogue of AE, three residues additional were parametrized using the same methodology applied to PET. These residues specifically represent the serine bonded to PET and two residues corresponding to the units subsequent to the cleavage of the connecting ester bond. The parametrization procedure involved quantum mechanics calculations conducted at the HF/6-31G* level using Gaussian 09.^40^ This was undertaken to establish generalized AMBER force field (GAFF)^41^ parameters for both the terminal and central units. Subsequently, restrained electrostatic potential (RESP) calculations^42,43^ were executed through the antechamber tool^44,45^ available in AmberTools 21.^46^ This step resulted in initial partial charges, which were then redistributed using prepgen to achieve a zero net charge for each PET unit.

### Preparation of Input Structures

**Figure 2:**
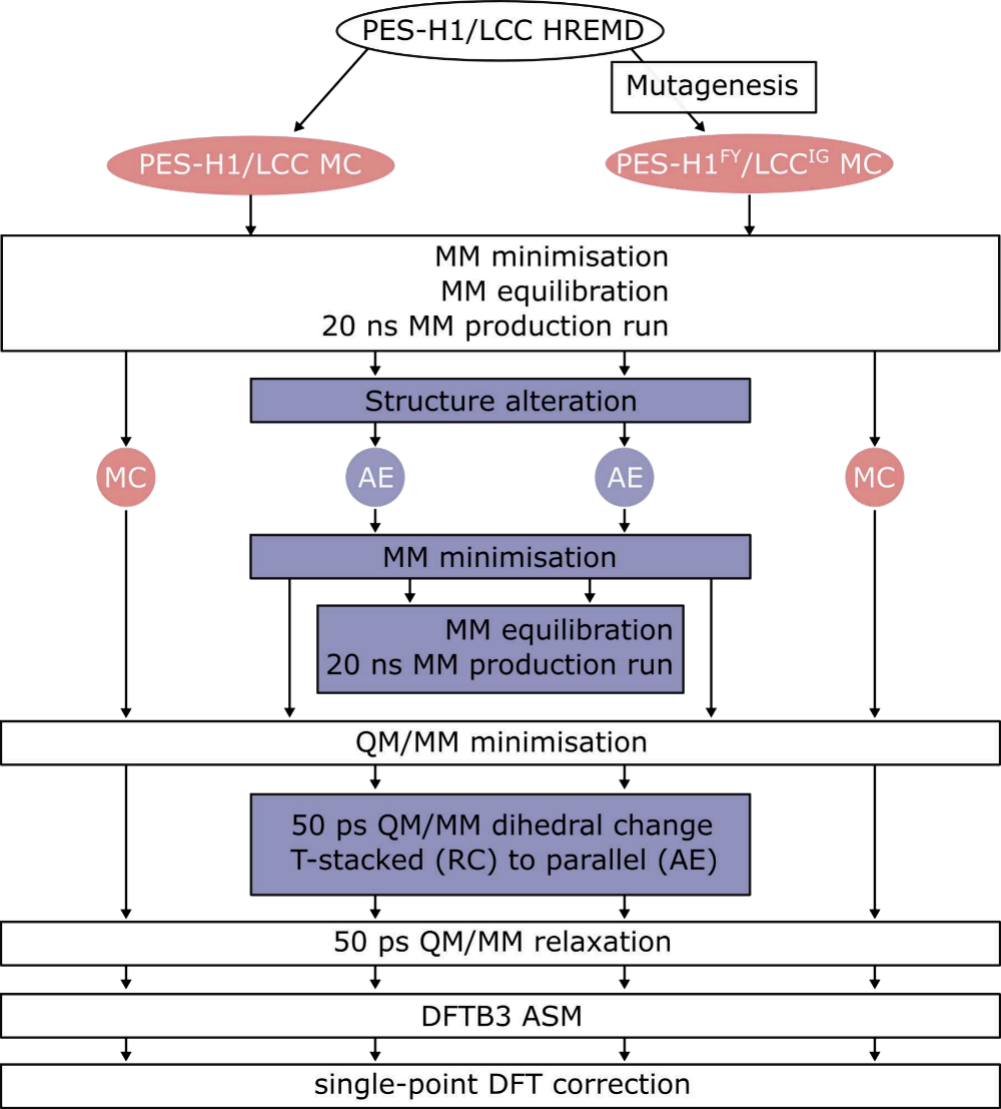
Schematic methodological workflow applied in this study.

The methodological workflow explained in the following is highlighted in Figure 2.

For both enzymes PES-H1 and LCC, the conformation with the catalytic serine on the *si*-side of the PET chain and the minimum distance between the PET ester carbon and the γ oxygen of this serine were extracted from previous Hamiltonian replica exchange molecular dynamics (HREMD) simulations using PDB 7CUV (chain B) for PES-H1 and PDB 4EBO for LCC.^14^ These starting structures consist of a PET molecule with five units plus the enzyme. The starting structures for the variants PES-H1^FY^ and LCC^IG^ were generated by introducing the corresponding mutations into these starting structures using PyMOL.^47^ The systems were solvated and neutralized by the addition of TIP3P water and sodium or chloride ions, respectively, using *tleap*. This resulted in ∼29,150 atoms for the PES-H1 systems and ∼28,500 atoms for the LCC systems. The enzymes were represented using the AMBER14SB force field, and parameters for the three PET unit types (i.e., two types of terminal and the central units) were incorporated using *tleap* as implemented in AMBER.^46^ The protonation states of titratable amino acids were modeled as predicted by Propka 3.4,^49,50^ resulting in a net charge of +5 for LCC and LCC^IG^ and −5 for PES-H1 and PES-H1^FY^ (see the Supporting Information for details).^14^ The energy of the solvated systems was minimized using the steepest descent algorithm^51^ followed by the conjugate gradient algorithm.^52^ Subsequent equilibration runs were performed at a temperature of 303 K and pressure of 1 bar in the NVT and NPT ensemble, utilizing the Langevin thermostat^53^ and the Berendsen barostat,^54^ respectively, with restraints applied to the positions of the protein and PET atoms. The MD production runs at the MM level spanned 20 ns in the NPT ensemble and included one-sided harmonic distance restraints to hold the PET ester in a productive pose. The one-sided harmonic restraint with a force constant of 20 kcal/mol/Å^2^, was initiated for a distance above 3.5 Å between the catalytic serine’s γ-oxygen and the PET’s ester carbon and for a distance above 3 Å between the PET’s ester carbonyl-oxygen and the center of mass of the backbone amine’s nitrogen of the oxyanion hole residues. This restraint becomes linear for distances above 10 Å. The SHAKE algorithm^55^ was used to constrain all bonds involving hydrogen atoms. A periodic box was employed for all runs. To reduce the computational demand, a cutoff at 8 Å was applied for the calculation of the short-range nonbonded interactions, and the particle mesh Ewald method^56,57^ was used to treat electrostatic interactions.

**Figure 3:**
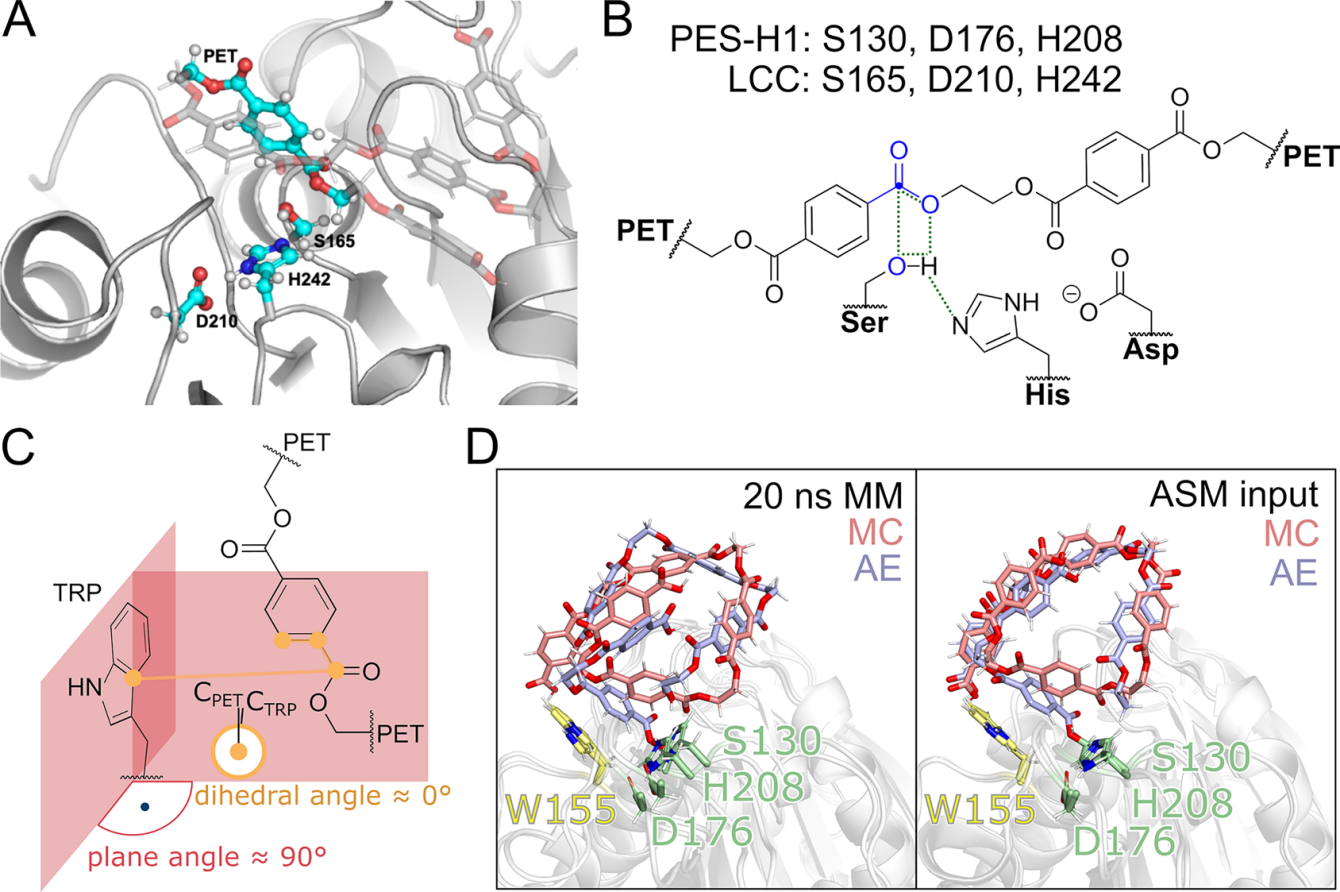
QM region and CVs employed in this study. (A) The QM region is shown (in licorice representation with C atoms in cyan, N in blue, O in red, and H in white) as embedded in the protein structure of LCC (shown as gray cartoon). (B) The QM region includes two PET units connected via the scissile ester bond and the side chains of the three catalytic triad residues (PES-H1: S130, D176, H208; LCC: S165, D210, H242). The distance CVs are highlighted in green, and the point-to-plane CVs are in blue, with the PET ester’s carbon corresponding to the point marked by a blue circle. The remaining atoms forming the plane include those in the ester between the PET units in the MC and the ester between PET and the catalytic serine in the AE, respectively. (C) An additional dihedral CV for dihedral angle θ is included to represent the angle between the tryptophan and PET ring planes (red). The dihedral θ (orange) and the ring plane angles (red) are highlighted schematically for the MC state preferring an approximate T-stacked orientation with a plane angle of ≈90°, which correlates with a dihedral angle θ of ≈0°. (D) PET conformations of the MC (red) and AE (blue) for PES-H1 after 20 ns MM production run (left) with prior two-step equilibration and the final structures after QM/MM preparation used as input for the ASM run (right).

The AE for each of the four systems was created from the reactant’s structure after the MM production run using PyMOL, retaining water molecules, ions, and box dimensions. The same protocol as for the reactants was followed including setup, minimization, MM equilibration, and 20 ns production run. Here, the distances describing the reaction and therefore used as collective variables (CVs) in the QM/MM simulations (see below) had to be restrained to keep the two PET units adjacent to the broken ester bond within the AE basin. Since the overall conformation of the long, flexible PET chain was altered due to conformational changes in the PET units other than the cleavage site during the MM production runs, the AE structure was subjected to the QM/MM preparation right after MM minimization. This involved a QM/MM optimization using steepest descent and conjugate gradient for both, reactant and AE. Only for the AE, a 50 ps QM/MM run was performed in the NVT ensemble applying a harmonic restraint to adjust the tryptophan–PET angle to the prevalent one during the MM production run. For the PES-H1^FY^ variant, this step was skipped as the preferred tryptophan–PET angle was already obtained after MM MD and was therefore already present after QM/MM optimization. The last step before the actual ASM run comprised an unrestrained 50 ps QM/MM relaxation in the NVT ensemble for both reactant and AE of each system. The QM region comprised 66 atoms and 5 link atoms for PES-H1 and PES-H1^FY^, and 64 atoms and 4 link atoms for LCC and LCC^IG^. It includes the side chains of the catalytic serine, histidine, and aspartate, as well as two PET units connected by the scissile ester bond (Figure 3 A and B). In the case of LCC/LCC^IG^, one of these units is the terminal one. It was described by the DFTB3 method without using the SHAKE algorithm, to allow for proton transfers.^58^ As nonbonded cutoff, 10 Å was used.

### ASM

The simulation of PET acylation (MC → AE, see Figure 1) was conducted using the ASM in connection with QM/MM with DFTB3 as QM method and AMBER14SB plus TIP3P for the MM region, as implemented in AMBER.^39,46^ The initial and final states for the pathway connecting the reactant and the AE in CV space were defined through QM/MM relaxation simulations. As CVs, eight variables were used: five distances describing the reaction, one dihedral angle θ representing the change in orientation of the PET ring, and two point-to-plane distances reflecting the hybridization state of the PET ester’s carbon to enhance convergence (Figure 3B and C). MD simulations were initiated from a total of 96 nodes, equidistantly positioned along the initial guess. Half of these simulations started from the reactant state, while the other half began from the AE state. The structures were brought to the corresponding node positions during 1 ps MD runs by gradually increasing the force constants of the biasing potential from zero to the target value automatically determined by ASM. Upon convergence of the string to the minimum free-energy path (MFEP), a path collective variable (path-CV) was defined to monitor the progress along this path and consequently, the advancement along the reaction. Umbrella sampling MD (USMD) simulations^59,60^ were then performed, restraining each replica to the corresponding node on the string for the US windows using an automatically defined potential. The potential of mean force (PMF) was obtained via umbrella integration (UI)^61−63^ and assumed to be converged when the 95% confidence interval at the TS fell below 1 kcal/mol.

### DFT Correction

The PMFs obtained from the ASM runs were corrected to the M06-2X/6-31+G(d,p) level of theory^64^ by applying a cubic splines correction based on single-point energy calculations for relaxed structures along the reaction path. The details of the method can be found in the original publication.^65^ Briefly, the selected structures were first optimized using the DFTB3 method, followed by single-point calculations using the M06-2X functional with the 6-31+G(d,p) basis set, employing the Gaussian 09 software.^40^ The energy difference (Δ*E* = *E*_DFT_ – *E*_DFTB3_) was then interpolated and subtracted from the original PMF values to obtain the corrected PMF. The method relies on the assumption that the MFEP at low and high levels of theory are sufficiently similar. This was checked by performing climbing-image nudged-elastic band (CI-NEB)^66^ calculations for the reaction process at the M06-2X/6-31+G(d,p)/MM level of theory and comparing the path to the MFEP obtained with ASM (Figure S4).

## Results and Discussion

### MC and AE Prefer Different PET Ring Orientations

**Figure 4:**
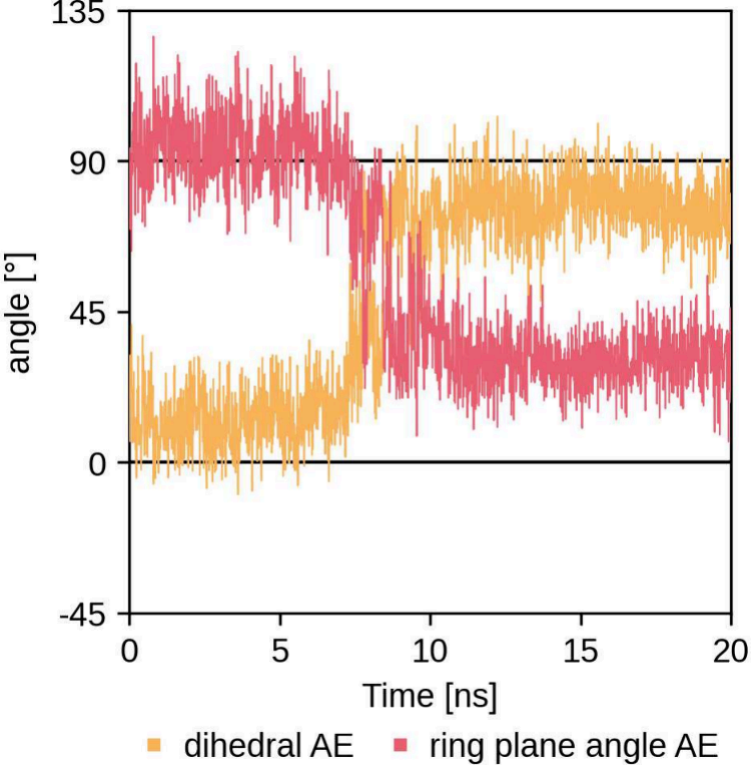
Dihedral angle θ (orange) and the angle between the tryptophan and PET ring planes (red) during the 20 ns MM production run of the AE of PES-H1.

To conduct QM/MM simulations of PET acylation, we extracted starting structures of PET–enzyme complexes from our previous Hamiltonian replica exchange MD simulations, which included a PET chain with 5 units and either LCC or PES-H1.^14^ The initial configurations for the LCC^IG^ and PES-H1^FY^ variants were obtained by introducing the corresponding mutations. Analysis of these binding poses revealed that the two PET units sharing the scissile ester bond are tightly bound, whereas the units further away exhibit looser interactions with the enzyme. The four resultant Michaelis complexes (MCs) were subjected to 20 ns MM simulations, which confirmed the stability of the chosen binding poses. However, the chain termini remained flexible (Figure S1). The final structures served as templates to manually generate AE structures using PyMOL, which were then also subjected to 20 ns MM simulations, with restraints being applied to prevent the leaving group from diffusing away from the AE. Two notable findings emerged: First, the flexibility of the loosely bound PET units further away from the scissile ester bond resulted in conformations unsuitable for subsequent QM/MM simulations using the ASM due to their difference to the MC conformations (Figure 3D, left). Second, the benzene ring of PET bound to the catalytic serine in the AE demonstrated a preference for a parallel orientation with respect to the “wobbling” tryptophan, whereas the MC favored a T-stacked orientation. This transition from T-stacked to parallel π–π interactions was observed during MM minimization (PES-H1^FY^) and during the beginning (LCC, LCC^IG^) or middle (PES-H1) of the MM production run. This shift, previously discussed by Han et al.^20^ in the context of product liberation, was not specifically mapped to a reaction step by the authors. Our results suggest that this transition occurs during the acylation phase. To address these findings, we only conducted energy minimization for the AE structures at the MM level and then switched to the QM/MM level where we performed energy minimization and a 50 ps MD run with restraints to enforce the transition of the benzene ring from T-stacked to parallel. Since plane angles are not currently implemented as restraints or CVs in the ASM, we have identified a dihedral angle θ that correlates well with the plane angle change and can be used both as a restraint during structure preparation and as a CV during ASM reaction simulation (Figure 3C and 4). A dihedral angle θ of approximately 0° corresponds to a T-stacked orientation, while an angle of approximately 90° indicates a parallel orientation. After enforcing the preferred parallel plane–plane angle in the AE, both MC and AE structures were subjected to a 50 ps QM/MM relaxation without restraints before conducting the final ASM run. This approach resulted in MC and AE structures that were close enough to each other for the ASM, as the PET did not move as much in the QM/MM runs of the AE as it did in the MM runs (Figure 3D, right).

### PET Ring Orientation Change Correlates with Acylation

To perform QM/MM calculations, we used the final MC and AE structures after 50 ps QM/MM relaxation as input configurations (Figure S2). We employed the semiempirical DFTB3 method^58^ to describe the QM region, encompassing the side chains of the catalytic residues (LCC and LCC^IG^: S165, D210, H242; PES-H1 and PES-H1^FY^: S130, D176, H208) and the two PET units sharing the scissile ester (Figure 3). This QM method, successfully applied in previous studies in combination with ASM, offers a balanced trade-off between accuracy and computational demand.^16^

**Figure 5:**
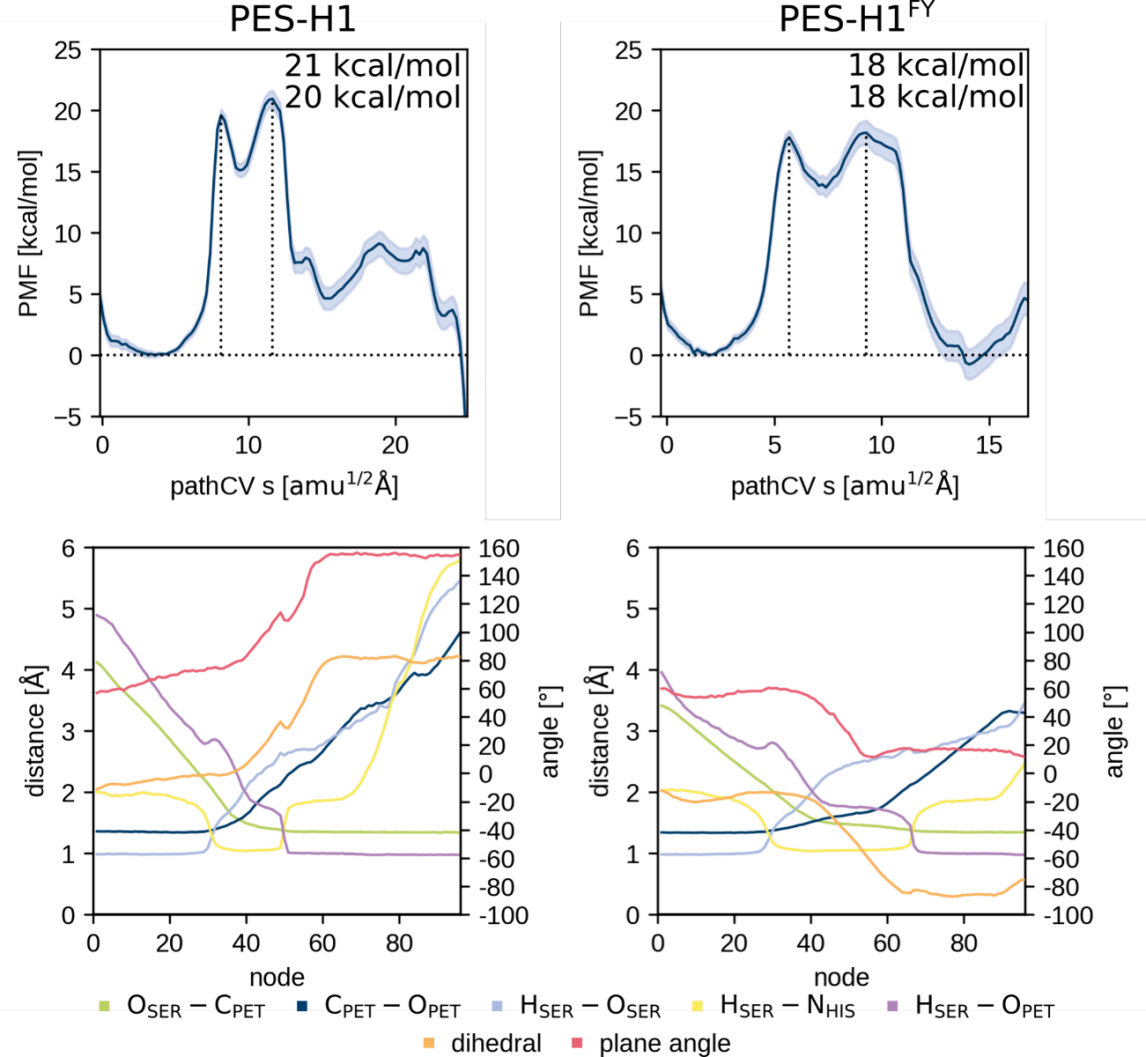
PMF of the PET acylation (MC → AE, see Figure 1) of PES-H1 (left) and its highly active variant PES-H1^FY^ (right). The height of the two barriers is provided as well. The lower plots show the corresponding averaged distance CV values per node during umbrella sampling of the converged string and, thus, depicts the CV progression along the reaction. Node 0 corresponds to the MC and node 96 to the AE state. Additionally, the angle between the ring planes of the “wobbling” tryptophan and the PET benzene moiety is plotted in red to highlight the correlation with the dihedral angle θ change in orange.

**Figure 6:**
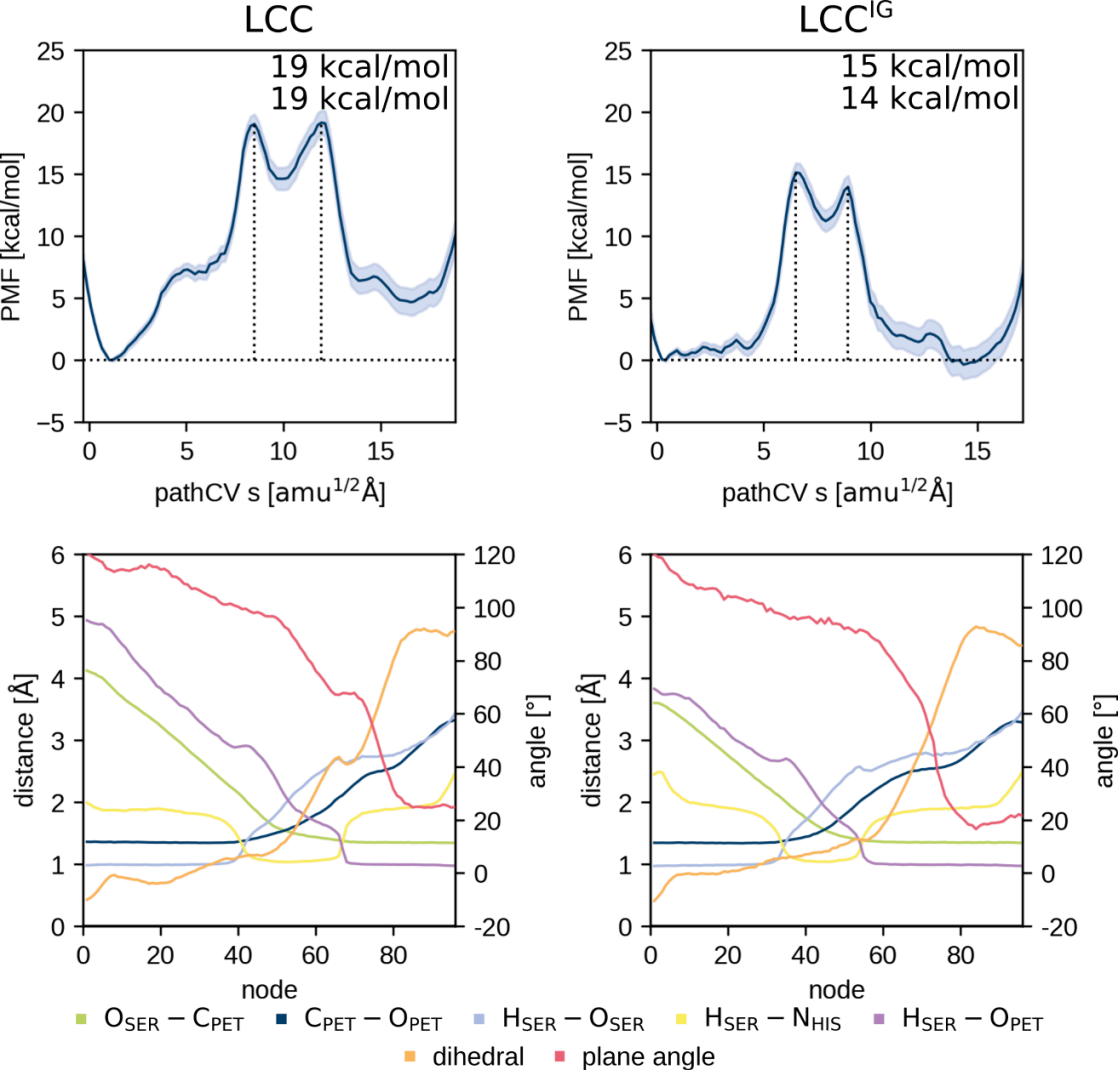
PMF of the PET acylation (MC → AE, see Figure 1) of LCC (left) and its highly active variant LCC^IG^ (right). The height of the two barriers is provided as well. The lower plots shows the corresponding averaged distance CV values per node during umbrella sampling of the converged string and, thus, depicts the CV progression along the reaction. Node 0 corresponds to the MC and node 96 to the AE state. Additionally, the angle between the ring planes of the “wobbling” tryptophan and the PET benzene moiety is plotted in red to highlight the correlation with the dihedral angle θ change in orange.

**Figure 7:**
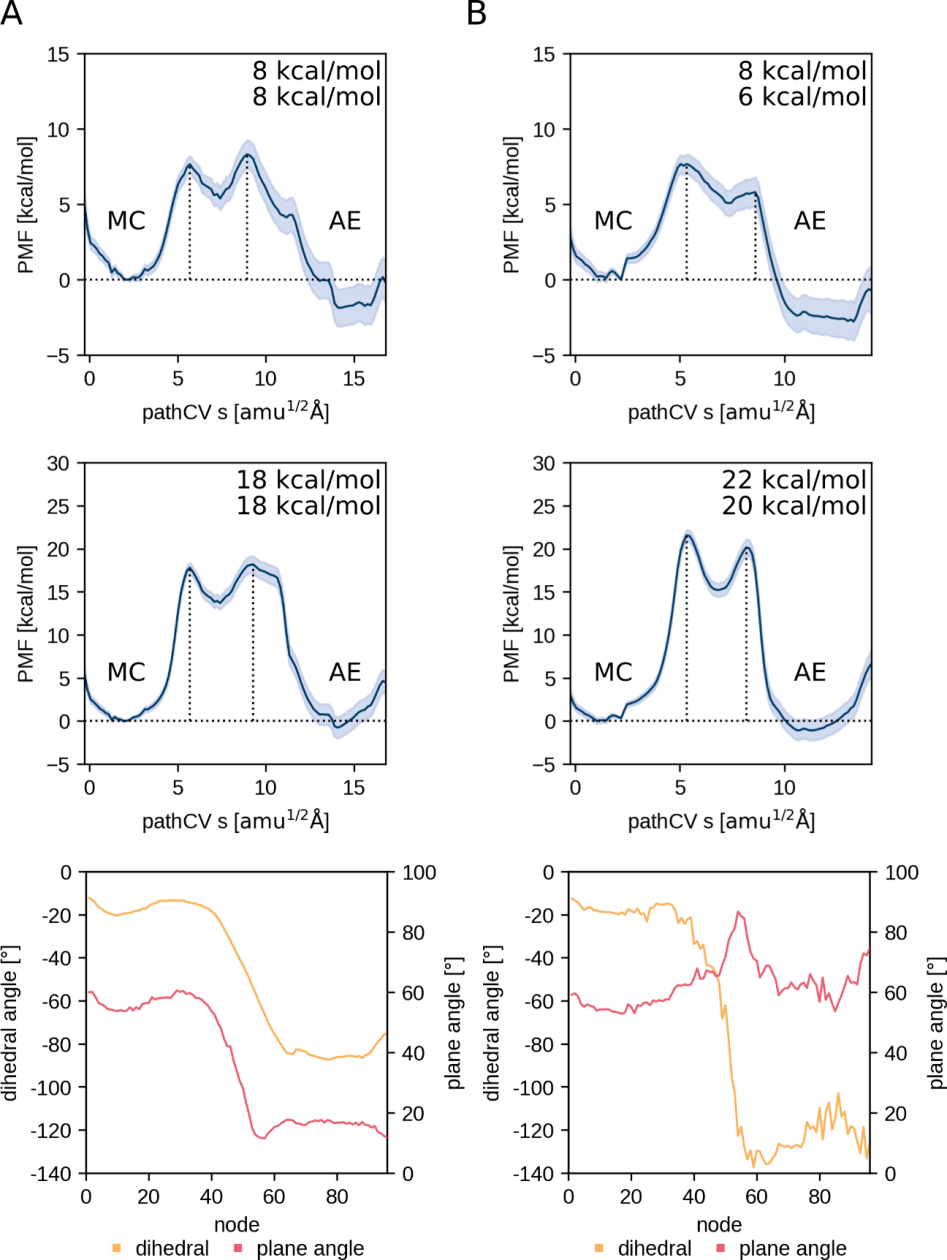
Comparison of the PMF using DFTB3 only (top) and after correction for DFT level (center), as well as the average dihedral and plane angles per node (bottom) for PES-H1^FY^ when incorporating the dihedral as a CV (A) and without the dihedral CV (B).

Additionally, we corrected the resulting PMF on DFT level using the M06-2X functional^64^ with the 6-31+G(d,p) basis set, which has proven effective in QM/MM studies of PET hydrolases.^15,17^ We used relevant distances depicting bond formations and breaking during the reaction as CVs, alongside two point-plane distances defining the hybridization state of the PET ester’s carbon, to enhance convergence.^16^ Furthermore, we included the dihedral angle θ as a CV to monitor the plane angle change. Average values for each CV, as well as the plane angle were calculated for each node after the ASM string was converged (Figure 5 and 6). In all cases, a smooth transition from a dihedral angle θ of approximately 0° (T-stacking) to approximately ±90° (parallel orientation) toward the ring plane of the “wobbling” tryptophan was revealed, showing good correlation with the plane angle change during the reaction. This smooth transition underscores the importance of including the plane angle change as a dihedral CV. As a proof of concept, we performed the same ASM calculation for PES-H1^FY^ without including the dihedral θ CV. In this case, the dihedral θ for MC and AE was not fixed, allowing for more flexibility and a mixture of parallel conformations adopted after 90° rotation and T-stacked conformations, which are adopted after 180° rotation, when approaching the product. Therefore, the PET ring atom defining the dihedral angle θ of this new T-stacked conformation points away from the “wobbling” tryptophan instead of toward it as in the MC, which explains the slight difference between the dihedral angle θ in the MC and the AE (Figure 7). Thus, the change in ring orientation happens even when it is not included as a CV, highlighting the link between this motion and the reaction. The progression of the distance CVs in combination with the PMFs describes the course of the reaction and enables mapping the ring orientation change to a specific step during the acylation reaction (Figure 5 and 6). The reaction mechanism matches that from previous works, suggesting a two-step acylation with the tetrahedral intermediate state as a shallow minimum on the free energy surface.^15,17,22−25,30,33^ First, the catalytic histidine abstracts a proton from the catalytic serine, resulting in TS1 (Figure 1). Then, the serine attacks the ester carbon, yielding the tetrahedral intermediate with its oxyanion stabilized by the oxyanion hole. While the converged MFEPs do not change significantly upon inclusion of the dihedral CV (see Figure S5), the mixing of states in absence of the dihedral CV affects the resulting PMFs. For instance, in case of LCC^IG^, the calculations fail to resolve the two transition states, resulting in an apparently single-step process.

The oxyanion is then converted back into a carbonyl oxygen by breaking the ester bond between the two PET units, generating a new oxyanion. The second transition state corresponds to the second proton transfer from the catalytic histidine to this oxyanion of the leaving group, liberating an alcohol and resulting in the AE. The steepest change in the plane angle and the dihedral angle θ begins with the formation of the tetrahedral intermediate, and in the case of LCC^IG^ after the second proton transfer, and is thus concerted with the acylation (Figure 5 and 6). Comparison of the PMFs of the ASM runs with and without the dihedral θ CV shows that the barriers in the DFT-corrected PMFs are somewhat larger when the dihedral θ CV is not included, suggesting that the conformational change of the plane angle during the reaction is energetically favorable, but this is neglected when the corresponding CV is omitted (Figure 7).

### QM Method and Choice of Collective Variables Influence the Energetics of the Reaction

Comparison of the uncorrected DFTB3 PMF profiles with those corrected by DFT (M06-2X/6-31G(d,p)) shows that the energy barriers are generally much lower for DFTB3, most likely due to an underestimation of the energy barrier for proton transfer with DFTB3 (Figures 7 and S3). In general, the choice of the QM method influences the PMF outcome, which is mirrored by the diverging results in previous works, suggesting either a one-step^16,26^ or a two-step^15,17,22−25,30,31,33^ mechanism for the acylation of PET hydrolases.

As an example, Boneta et al. used the AM1^67^ method to describe the QM region and corrected the resulting PMF on DFT level using the M06-2X functional^64^ with the 6-31+G(d,p) basis set, i.e. the same correction as applied in this study. This yielded a clear two-step PMF for LCC^ICCG^ and *Is*PETase, which may have been favored by AM1, as AM1 tends to overestimate energy barriers.^15^ On the other hand, QM/MM free energy profiles obtained using DFTB3, which tends to underestimate proton transfer barriers,^16^ indicate a one-step profile for the FAST-PETase^16^ and the *Is*PETase.^32^ Similarly, Jerves et al. postulated a one-step profile using the Perdew–Burke–Ernzerhof (PBE) functional,^68,69^ which also gives small energy barriers for proton transfers.^26,70,71^

The PMFs obtained here using the DFTB3 method also suggest that the reaction might be a one-step rather than a two-step mechanism (with either a very shallow or no significant minimum for the TI), but it is turned into a clear two-step profile upon correction to DFT (M06-2X) (Figures 7, S3, 5 and 6). Interestingly, when the dihedral angle controlling the orientation of the “wobbling” tryptophan is added as a CV, the apparent single step acylation in LCC^IG^ got resolved into a two-step process. This could further explain the single-step acylation observed in other studies,^16,26,32^ who did not consider coupling between the dihedral angle and the reaction coordinate. We therefore suggest a two-step mechanism including a metastable intermediate state for the acylation stage of PET degradation, which may appear as a one-step mechanism when using methods underestimating proton transfer energies such as DFTB3 and PBE, or incomplete inclusion of relevant degrees of freedom.

### Decreased Acylation Barrier As One Reason for Increased Activity of Variants LCC^IG^ and PES-H1^FY^

In our previous study, the effect of single residues on the entry of PET into the active site of LCC and PES-H1 and their high-activity variants LCC^ICCG^ (represented by LCC^IG^) and PES-H1^FY^ was analyzed.^14^ The free energy surfaces of a PET chain moving into the active site suggested that the mutated residues promote PET entry by facilitating an unhindered entry and by stabilizing the bound over the unbound PET state. Our current results further show that both variants exhibit significantly lower activation free energy barriers for acylation (considering a 95% confidence interval of ≈1 kcal·mol^–1^; Figure 5 and 6). The MC of the variants has the same or a higher energy than the corresponding AE in the corrected PMF, while it exhibits a lower energy in the WT profiles. This indicates either a destabilization of the MC or a stabilization of the AE upon mutation. A destabilization of the MC would explain the reduced activation free energy barrier with respect to the MC, while stabilization of the AE would imply enhanced stabilization of the TSs. However, in our previous study involving a free energy analysis of PET binding, we identified a stabilization of the MC upon mutation for all mutation sites in the variants LCC^IG^ and PES-H1^FY^.^14^ It is worth pointing out that the positions of the TSs in the CV space do not differ significantly between the variants (see Figure S6). This indicates that the differences in the environment only change the energy profile of the reaction, without altering the geometry of the TSs. We therefore conclude that stabilization of the TS and AE (with respect to the MC) is the cause of the changes in the PMF and the reduction of the activation free energy barrier, which promotes acylation and potentially also the PET-degradation activity of the variants, as experimentally established.^5,13^

## Conclusion

This study explored the initial chemical step of enzymatic PET degradation, the acylation process, in detail, using QM/MM MD sampling with the adaptive string method (ASM). We focused on two well-studied PET hydrolases, LCC and LCC^ICCG^ (represented here by LCC^IG^), alongside PES-H1 and PES-H1^FY^.^5,13^ Our investigation revealed two distinct conformations of the PET benzene ring, facilitating different types of π–π interactions with the “wobbling” tryptophan residue in the MC (T-stacked) and AE (parallel). This conformational shift appears to occur concertedly with acylation and contributes to the resulting free energy profile. Therefore, it is crucial to consider the varying orientations of the PET benzene ring during QM/MM simulations (e.g., by including a CV describing this during sampling), an aspect previously overlooked. Furthermore, we found that acylation proceeds through a two-step process involving a metastable tetrahedral intermediate, which is potentially missed in other studies due to the QM method employed. Additionally, both high-activity variants exhibited reduced activation free energy barriers compared to their respective WT enzymes. Interestingly, in the variants, the MC and AE states were energetically equivalent, whereas the MC is more stable in the WT enzymes. We hypothesize that this relative stabilization of the AE is coupled with the stabilization of the transition state, contributing to enhanced activity. However, we assume that the reduction of the energy barrier by approximately 2–5 kcal/mol is not the only factor driving the substantial activity increase. The multifaceted nature of PET enzyme activity is influenced by various mechanisms such as substrate adsorption, entry to the binding site, productive substrate binding, and product liberation, in addition to the catalysis of the chemical process of substrate conversion addressed here. Consequently, comprehending the heightened PET-degradation activity of LCC^ICCG^ and PES-H1^FY^ demands a comprehensive approach encompassing all pertinent mechanisms.

## Data Availability

Supporting Information includes the topology and coordinates (in Amber format) of MC and AE structures used for ASM calculations for all the variants. It also includes PDB files of the structures used to generate Figures 3D and Figures S1 and S2. The topology files, relaxed structures, input files for ASM calculations and resulting PMFs are available on Zenodo (https://zenodo.org/doi/10.5281/zenodo.13326292).

## Supporting Information Available

Protonation states of titratable residues, figures of PET conformations after 20 ns MM production run and after 50 ps QM/MM relaxation, and PMFs obtained at the DFTB3/ff14SB level (PDF)
