# Supplementary material for: Influence of Wobbling Tryptophan and Mutations on PET Degradation Explored by QM/MM Free Energy Calculations
Source: J Chem Inf Model. 2024 Sep 30;64(19):7544–54. doi: 10.1021/acs.jcim.4c00776 (PMC11480989; doi:10.1021/acs.jcim.4c00776)
Supplement: Supplementary file 1 — ci4c00776_si_001.pdf [file ci4c00776_si_001.pdf]

Supplementary Information

Influence of Wobbling Tryptophan and Mutations on PET Degradation Explored by QM/MM Free Energy Calculations

Anna Jäckering,[†,‡] Marc van der Kamp,[¶] Birgit Strodel,[*,†,‡] and Kirill Zinovjev[*,§]

†*Institute of Theoretical and Computational Chemistry, Heinrich Heine University, Düsseldorf, Universitätsstr. 1, 40225 Düsseldorf, Germany*

‡*Institute of Biological Information Processing: Structural Biochemistry (IBI-7), Forschungszentrum Jülich, Wilhelm-Johnen-Straße, 52428 Jülich, Germany*

¶*School of Biochemistry, University Walk, University of Bristol, Bristol BS8 1TD, United Kingdom*

§*Departament de Química Física, Universitat de València, 46100 Burjassot, Spain*

E-mail: b.strodel@fz-juelich.de; kirill.zinovjev@uv.es

Protonation states of titratable residues

PES-H1 and PES-H1$^{\mathbf{FY}}$:

- All Arg (5, 18, 30, 74, 90, 97, 99, 109, 115, 117, 122, 157, 164, 203, 227, 234, 252) and Lys (158, 197, 219, 226) positively charged.
- All Glu (4, 11, 15, 66, 146, 185, 201, 236, 250) and Asp (8, 34, 46, 83, 92, 95, 106, 119, 175, 194, 196, 214, 230, 231, 232, 244, 245) negatively charged.
- His neutral: His protonated on Nϵ (107, 128, 155, 183), His protonated on NDδ (207).
- Overall charge: -5, 5 Na^+ ions added to neutralize.

LCC and LCC$^{\mathbf{IG}}$:

- All Arg (6, 12, 30, 72, 73, 89, 96, 108, 116, 118, 123, 138, 234, 236, 251, 255) and Lys (147, 159, 197, 226) positively charged.
- All Glu (141, 173, 201) and Asp (18, 63, 91, 94, 120, 158, 175, 203, 230, 232, 244, 249) negatively charged.
- His neutral: His protonated on Nϵ (77, 129, 156, 183, 256), His protonated on Nδ (207).
- Overall charge: $+5$, 5 Cl^- ions added to neutralize.

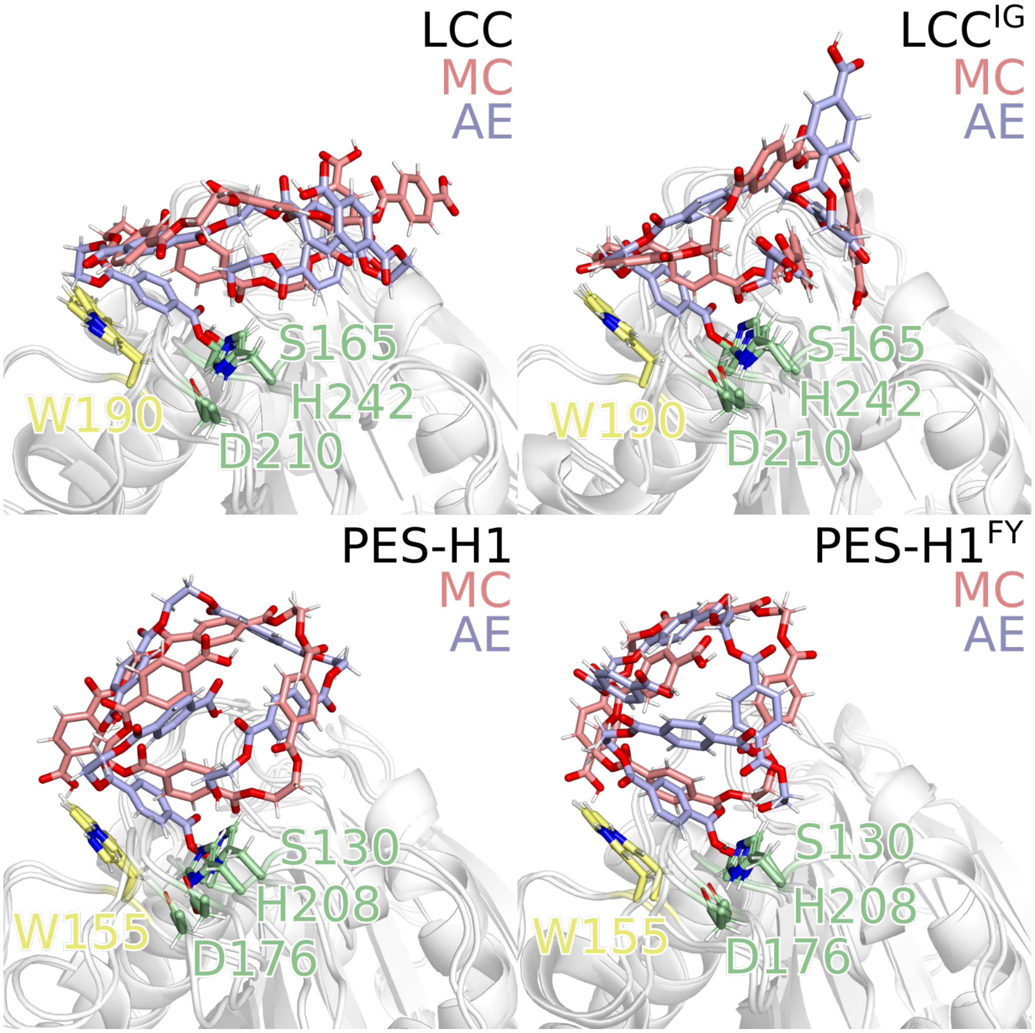

Figure S1: PET conformations of the MC (red) and AE (blue) after 20 ns MM production run for LCC, LCC^{IG}, PES-H1 and PES-H1^{FY}.

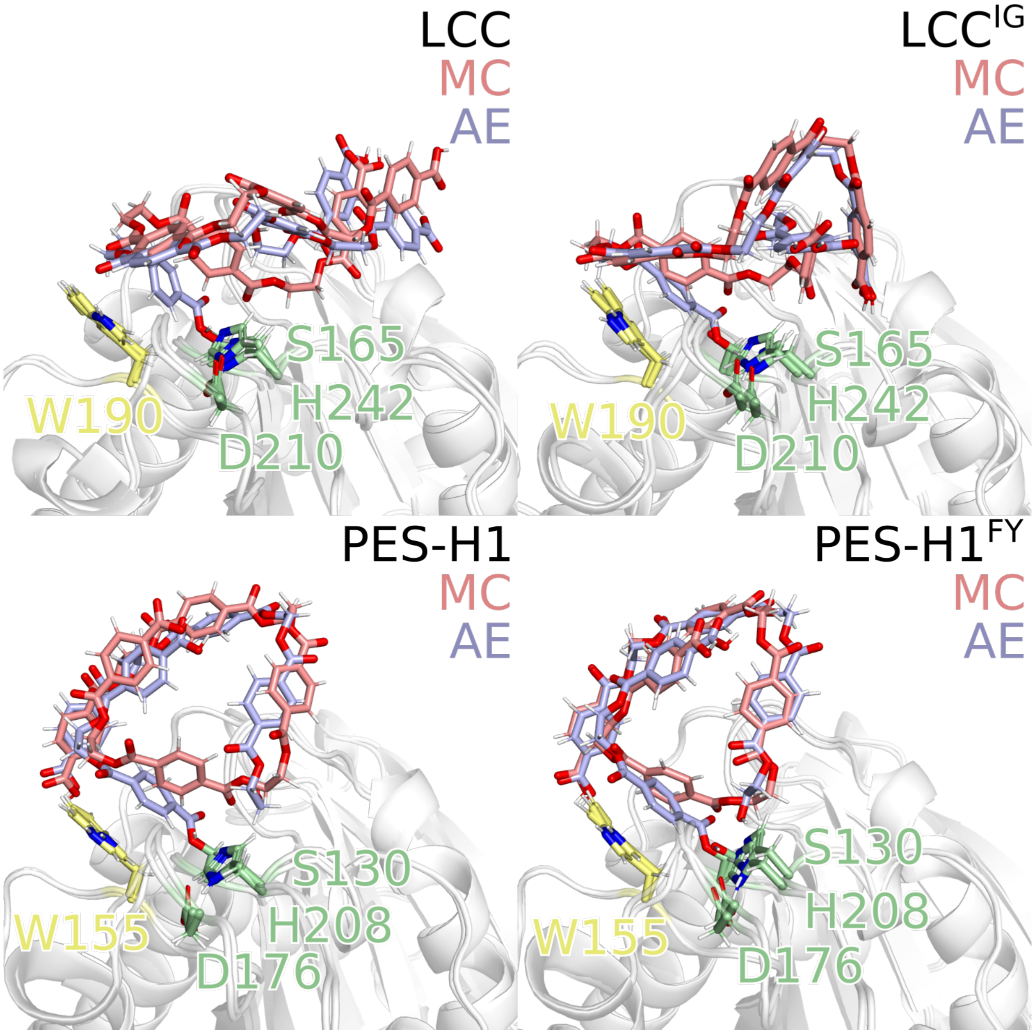

Figure S2: PET conformations of the MC (red) and AE (blue) after 50 ps QM/MM relaxation before conducting ASM for LCC, LCC^{IG}, PES-H1 and PES-$H1^{FY}$.

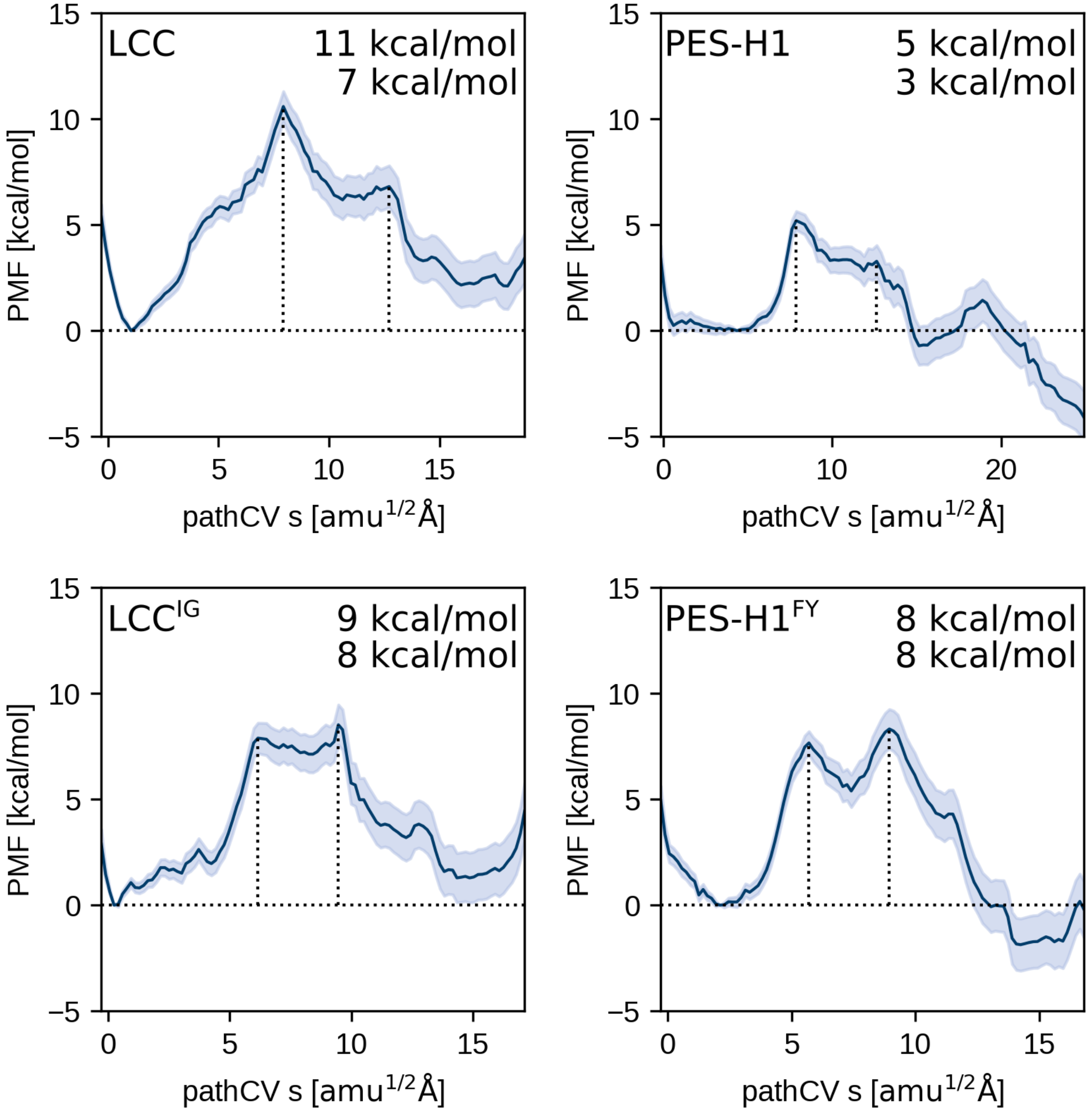

Figure S3: PMF at the DFTB3/ff14SB level of the PET deacylation of LCC (top, left) and PES-H1 (bottom, right) and their highly active variants LCC^{IG} (top, right) and $PES\text{-}H1^{FY}$ (bottom, right). The height of the two barriers is provided as well.

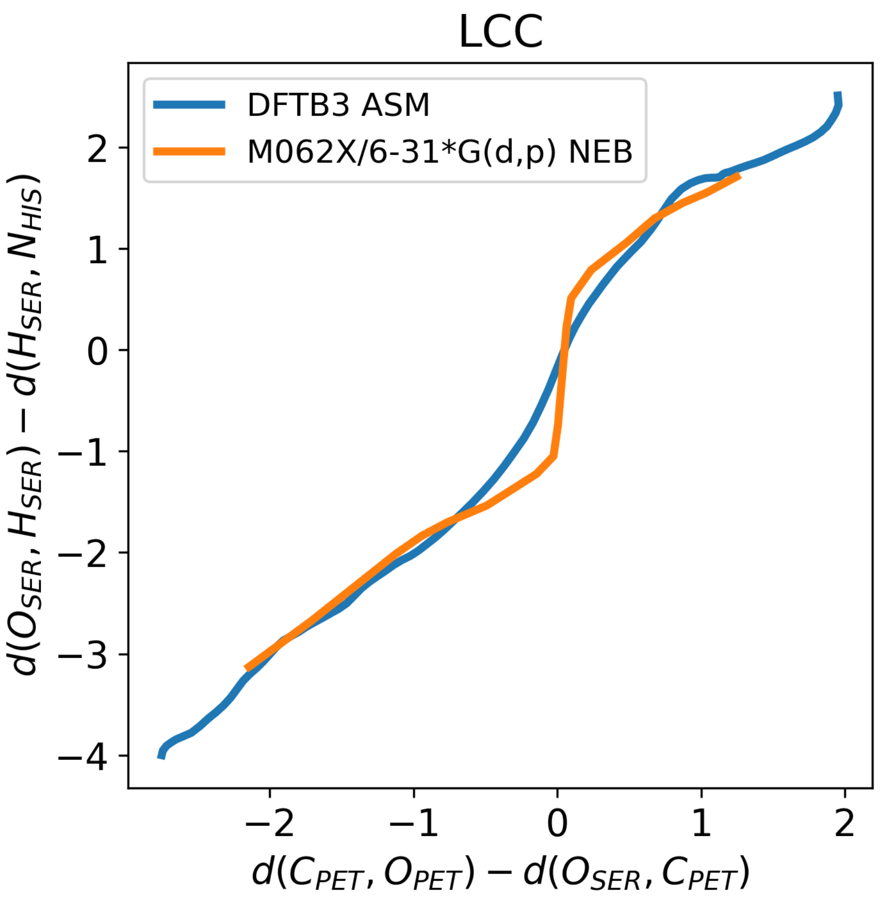

Figure S4: Comparison between DFTB3/MM ASM MFEP and M06-2x/6-31G*(d,p)/MM CI-NEB MEP.

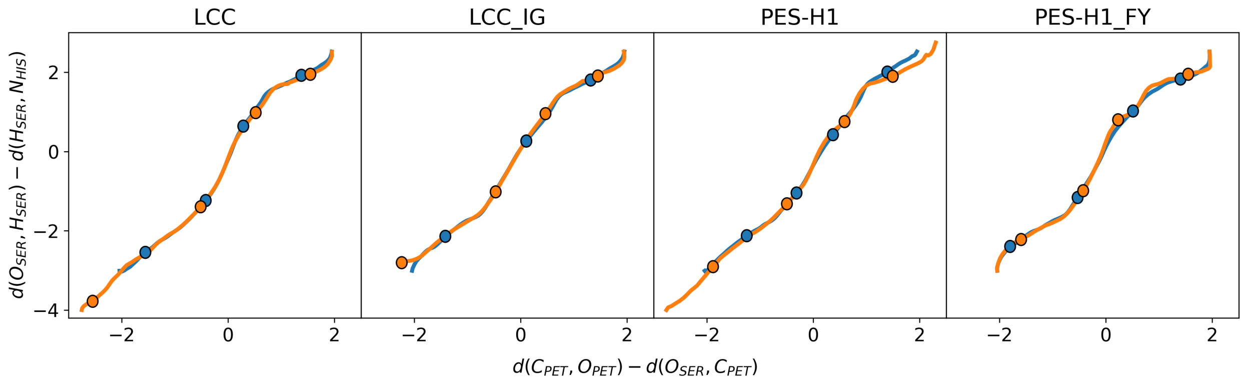

Figure S5: ASM MFEPs obtained with (orange) and without (blue) dihedral CV. The reactants, two TSs and products are depicted as circles. In case of LCC_IG without dihedral CV only one TS was found.

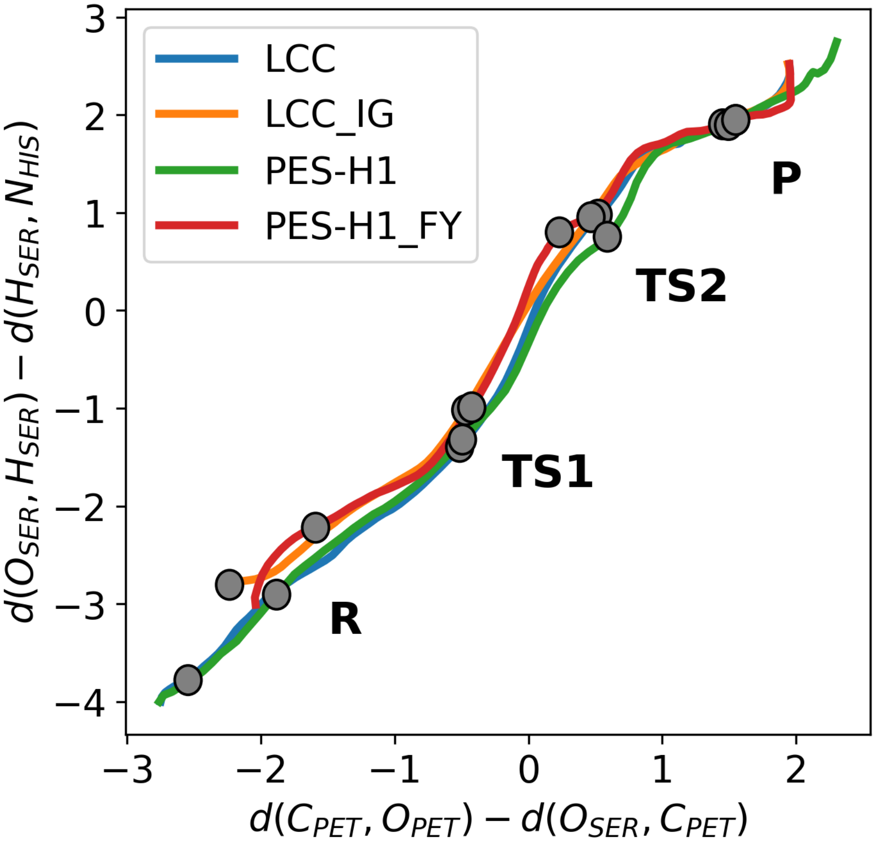

Figure S6: ASM MFEPs and stationary points for four variants studied.
